# Tract Specific White Matter Lesion Load Affects White Matter Microstructure and Their Relationships With Functional Connectivity and Cognitive Decline

**DOI:** 10.3389/fnagi.2021.760663

**Published:** 2022-02-02

**Authors:** Tae Kim, Howard J. Aizenstein, Beth E. Snitz, Yu Cheng, Yue-Fang Chang, Rebecca E. Roush, Theodore J. Huppert, Annie Cohen, Jack Doman, James T. Becker

**Affiliations:** ^1^Department of Radiology, University of Pittsburgh, Pittsburgh, PA, United States; ^2^Department of Bioengineering, University of Pittsburgh, Pittsburgh, PA, United States; ^3^Department of Psychiatry, University of Pittsburgh, Pittsburgh, PA, United States; ^4^Department of Neurology, University of Pittsburgh, Pittsburgh, PA, United States; ^5^Departments of Statistics and Biostatistics, University of Pittsburgh, Pittsburgh, PA, United States; ^6^Department of Neurosurgery, University of Pittsburgh, Pittsburgh, PA, United States; ^7^Deparement of Electrical and Computer Engineering, University of Pittsburgh, Pittsburgh, PA, United States

**Keywords:** white matter fiber tracts, Alzheimer’s disease, cognitive impairment, aging, white matter hyperintensity (WMH), white matter lesion (WML), functional connectivity (FC)

## Abstract

White matter hyperintensities (WMHs) are associated with cognitive decline. Assessing the effect of WMH on WM microstructural changes and its relationships with structural and functional connectivity to multiple cognitive domains are helpful to better understand the pathophysiological processes of cognitive impairment. 65 participants (49 normal and 16 MCI subjects, age: 67.4 ± 8.3 years, 44 females) were studied at 3T. The WMHs and fifty fiber tracts were automatically segmented from the T1/T2-weighted images and diffusion-weighted images, respectively. Tract-profiles of WMH were compared with those of apparent fiber density (AFD). The relationship between AFD and tract connectivity (TC) was assessed. Functional connectivity (FC) between tract ends obtained from resting-state functional MRI was examined in relation to TC. Tract-specific relationships of WMH, TC and FC with a multi-domain neuropsychological test battery and Montreal Cognitive Assessment (MoCA) were also separately assessed by lasso linear regression. Indirect pathways of TC and FC between WMH and multiple cognitive measures were tested using the mediation analysis. Higher WMH loads in WM tracts were locally matched with the reduced AFD, which was related to decrease in TC. However, no direct relationship was found between TC and FC. Tract-specific changes on WMH, TC and FC for each cognitive performance may explain that macro- and microstructural and functional changes are associated differently with each cognitive domain in a fiber specific manner. In these identified tracts, the differences between normal and MCI for WMH and TC were increased, and the relationships of WMH, TC and FC with cognitive outcomes were more significant, compared to the results from all tracts. Indirect pathways of two-step (TC-FC) between WMH and all cognitive domains were significant (*p* < 0.0083 with Bonferroni correction), while the separated indirect pathways through TC and through FC were different depending on cognitive domain. Deterioration in specific cognitive domains may be affected by alterations in a set of different tracts that are differently associated with macrostructural, microstructural, and function changes. Thus, assessments of WMH and its associated changes on specific tracts help for better understanding of the interrelationships of multiple changes in cognitive impairment.

## Introduction

White matter lesions appearing as white matter hyperintensities (WMH) on T_2_-weighted magnetic resonance imaging (MRI) are often observed even in the brains of cognitively normal older adults (Meyer et al., 1992; Ylikoski et al., 1995). However, as WMH burden increases, it is likely to become associated with cognitive decline (Debette and Markus, 2010; Prins and Scheltens, 2015). The WMH may reflect the macrostructural damage to white matter (WM) and may disrupt microstructural features in tracts traversing the lesions. They may influence interconnections among multiple regions of the brain taking place through WM pathways, and these may be associated with cognitive changes. Therefore, the effect of WMH on microstructural WM changes and their relationship with functional changes should be explained for the assessment of cognitive impairments.

The WM of the brain consists of bundles of myelinated fibers, known as fascicles or fiber tracts. The local microstructural alterations in the tracts associated with WMH can be detrimental to the connectivity of entire tract, which may interrupt the cortical to cortical (and/or subcortical) connections that could result in alterations of brain function (O’Sullivan et al., 2001). On the other hand, structural disruption does not necessarily represent functional disconnection (Batista-García-Ramó and Fernández-Verdecia, 2018). Thus, the direct relationship between WM connectivity of each individual tract and functional connectivity of the brain regions connecting that tract is necessary. In addition, since the mechanisms of structural and functional changes may be different, they may develop through different pathways of deterioration with different brain reserve. Thus, they may manifest differently for cognitive alterations progress. Therefore, the structural and functional connectivity could be differently related to various cognitive domains.

The deterioration of certain brain regions may affect certain cognitive functions, i.e., alterations in specific WM fiber-tracts may contribute to decreased performance in the specific cognitive domains (e.g., executive function) (Carnevale et al., 2018). For example, MD changes in the anterior WM correlated with selective loss of executive function in the elderly (Carnevale et al., 2018). Thus, the local microstructural changes due to WMH can play an important role in a chain of relationships between regional structural and functional connectivity and specific cognitive deficits. Identifying the relationships of these tract-specific characteristics with performance in each cognitive domain can provide better understanding of the inter-relationship of the multiple pathological changes that occur in the elderly leading to cognitive changes.

In this study, the effects of WMH on major WM pathways were assessed by a tract-based approach. We hypothesized that the relationships among WMH, fiber density, structural connectivity and functional connectivity were highly correlated, and these changes are associated with cognitive performance in a specific neuropsychological measure. Subsequently, we tested significances in the causal relationships between WMH and multiple cognitive measures mediated by microstructural and functional connectivity.

## Materials and Methods

### Participants

65 participants (ages: 67.4 ± 8.3 years, 44 females), 49 normal controls and 16 MCIs with comparable age and sex were studied at 3T Siemens Prisma (Erlangen, Germany) using a 64-channel head coil. All studies were approved according to the University of Pittsburgh Institutional Review Board and written informed consent was obtained from all participants. The individuals enrolled in the study did not have a self-reported history of major central nervous system pathology (e.g., epilepsy, tumor, Huntington’s disease, Parkinson’s disease, multiple sclerosis, stroke, head injury with loss of consciousness for more than 30 min), major psychiatric disease (e.g., severe major depression, bipolar disorder, substance abuse), or active cancer. We also excluded persons who had sensory or motor deficits sufficient to impair their ability to perform the core neuropsychological tasks. Individuals taking psychoactive medications at doses that could affect the neuropsychological testing or brain functional responses such as benzodiazepines (e.g., lorazepam) or narcotic analgesics (e.g., acetaminophen/propoxyphene) were likewise excluded from the study.

### Cognitive Assessments

Participants completed a multi-domain neuropsychological test battery, including tests of *memory* (Consortium to Establish a Registry of Alzheimer’s Disease Word List Learning test; modified Rey Osterrieth figure recall); *language* (30-item Boston Naming Test; verbal fluency); *attention* (Trail Making Test A; digit span forward); *executive functions* (Trail Making Test B; digit span backward; clock drawing); and *visuospatial construction* (modified Block Design; Rey Osterrieth figure copy). The Montreal Cognitive Assessment (MoCA) was also administered for a global screening measure.

### Magnetic Resonance Imaging Data Acquisition

MRI data was obtained by using part of the HCP imaging protocols.^1^ Anatomical images were acquired using a 3D T_1_-weighted magnetization prepared rapid gradient echo (MPRAGE; TR = 2,400 ms, TE = 2.22 ms, TI = 1,000 ms, FA = 8°, voxel size = 0.8 × 0.8 × 0.8 mm^3^) with the generalized autocalibrating partially parallel acquisition (GRAPPA) acceleration factor = 2. 2D T_2_-weighted fluid attenuation inversion recovery (FLAIR) images were acquired with TR = 9,690 ms, TE = 91 ms, TI = 2,500 ms, voxel size = 0.8 × 0.8 × 1.6 mm^3^, number of slices = 104 with no gaps, and GRAPPA factor = 2.

A total of four runs of diffusion MRI were acquired using simultaneous multislice (SMS) spin-echo EPI with TR = 3,230 ms, TE = 89.20 ms, voxel size = 1.5 × 1.5 × 1.5 mm^3^, number of slices = 92 with no gaps, multiband acceleration factor = 4; both 98 and 99 diffusion-weighted directions were acquired twice with reversed phase encoding direction (anterior-posterior (AP) and posterior-anterior (PA)) in order to correct for the EPI geometric distortion. A monopolar Stejskal-Tanner diffusion scheme was used with *b*-values of 0, 1,500, and 3,000 s/mm^2^ (Stejskal and Tanner, 1965).

Four runs (two sets of opposite phase encoding directions, AP-PA) of resting-state functional MRI (rs-fMRI) data were obtained using a 2D single-shot SMS gradient-echo echo-planar imaging (GE EPI; TR = 800 ms, TE = 37 ms, FOV = 208 × 208 mm^2^, voxel size = 2 × 2 × 2 mm^3^, FA = 52°, multiband acceleration factor = 8 and 72 slices with 420 volumes). Two spin-echo EPI acquisitions were acquired with opposite phase-encoding directions to calculate a spin-echo fieldmap, which was used for the correction of rs-fMRI geometric distortion. These spin-echo EPI images have the same geometrical, echo spacing, and phase encoding direction parameters as the GE-EPI rs-fMRI scans.

### Data Processing

The MRI data was processed using AFNI (v17.2.07),^2^ FSL (v6),^3^ and Mrtrix3^4^ programs combined with an in-house Matlab program (2017b MathWorks, Natick, MA).

#### White Matter Hyperintensity Segmentation

The T_1_-weighted images underwent: bias field correction, removal of gradient non-linearity and readout distortion, and alignment to the FLAIR images. The WMH regions were automatically segmented from two different contrasts of FLAIR and T_1_-weighted images using a deep learning algorithm based on deep fully convolutional network [TensorFlow (v1.8) using Keras] and ensemble models (Li et al., 2018; Kuijf et al., 2019). The binary WMH masks were transformed to the Montreal Neurological Institute (MNI) space for each subject. The summation of the number of voxels detected as WMH multiplied by voxel dimensions yields the total WMH volume (units of mm^3^) for each subject.

#### Processing Apparent Fiber Density and Tract Connectivity

The preprocessing of the DWI included: denoising (Veraart et al., 2016), eddy-current, motion, bias field, and gradient-non-linearity corrections. Then, the data were registered to the anatomical images, and then the images and diffusion vectors were linearly transformed to MNI space. Intensity normalization was also applied. These were processed by the HCP preprocessing pipelines.^5^

The fiber orientation distribution function (fODF) was obtained by high angular resolution diffusion imaging (HARDI) to overcome the non-specificity of FA by resolving complex fiber architecture, especially in WMH region (Jeurissen et al., 2014). The fiber orientation distribution (FOD) within each voxel was computed by using multi-shell multi-tissue constrained spherical deconvolution (CSD) with group-averaged WM/GM/CSF response functions, which enables the direct comparison of FOD amplitudes across subjects (Tournier et al., 2004, 2007). The response functions were acquired from 30 control subjects using the Dhollander algorithm (Dhollander et al., 2018). We confirmed that none of the WMH voxels were involved in the assessment of the WM response function. The absolute amplitude of each FOD can be directly associated with an apparent fiber density (AFD), which refers to a specific fiber population within a voxel containing multiple fiber orientations (Dell’Acqua and Tournier, 2019). The AFD was computed from the FOD, and total AFD was calculated by summing the fiber population within each voxel (Raffelt et al., 2012).

Fifty white matter fiber tracts were automatically determined by TractSeg (v2.1), which is calculated by a fully convolutional neural network using extracted FOD peaks that identify distinct orientation of each voxel (Wasserthal et al., 2018). Segmentations of the start/end regions of bundles, bundle segmentations, and tract orientation maps (TOMs) were acquired (Wasserthal et al., 2018). Bundle-specific tractography was automatically obtained by probabilistic tracking on the estimated TOMs (Wasserthal et al., 2019). The fifty tracts are listed in Supplementary Table 1.

To evaluate the AFD values along each tract, profiles were calculated by averaging the AFD in each segment for 100 equally distant centroid segments/points along the streamline (Wasserthal et al., 2020). The tract-profile of WMH was also obtained from the WMH masks along each tract with the same manner.

The connectivity strength of each segmented tract [referred as tract connectivity (TC)] was quantified as a cross-sectional area of the bundle that was calculated by the total AFD volume of the pathway of interest divided by the length of the bundle (taken as the mean streamline length). Thus, this is independent of fiber length.

#### The Comparison of White Matter Hyperintensitie and Apparent Fiber Density Profiles

To compare local differences in WMH profiles with corresponding changes in AFD profiles, we divided groups according to the amount of WMH volume: lower-WMH and higher-WMH group. The lower and higher WMH groups were defined as the bottom and top 30% of WMH volume from the normal controls, respectively (*n* = 15 in each group). Most MCI subjects have a large amount of WMH. MCI-WMH group was selected for MCI subjects with WMH volumes greater than 5 standard deviations from the mean of the lower-WMH group (Supplementary Figure 1). The AFD tract-profile of the lower- and higher-WMH control and MCI-WMH groups were compared to estimate the WMH effect on AFD in tract. The *t-*statistic for each segment/point in the AFD tract-profile was calculated between lower- and higher-WMH groups and between lower-WMH and MCI-WMH groups to identify local changes in the tract.

Note that this group classification is used only for the comparison of WMH and AFD tract profiles. For all other data processing, all 65 subjects were used.

#### Resting-State fMRI Data Processing

We followed the HCP data processing pipelines (Glasser et al., 2013). Briefly stated, gradient non-linear distortion correction was performed by the HCP-gradunwarp package using the scanner’s gradient coefficient file; EPI distortion was corrected by using FSL’s topup^6^ with a pair of spin-echo EPIs with opposite phase encoding directions that result reversed geometric distortion; motion was corrected by registering fMRI time-series images to the single-band reference image of the SMS acquisition. A demeaned and linearly detrended 12 motion parameters (translation, rotation and their derivatives of x, y, z axes) are provided as nuisance regression. High-resolution anatomical images and EPI data were normalized to MNI152 template space. Four sessions of rs-fMRI studies were concatenated. An independent component analysis (ICA) was used to find the spatial and temporal components of the functional networks (Beckmann et al., 2005) after 2000s FWHM high-pass temporal filtering for detrending-like behavior. Then, FSL’s FIX was used to automatically classify ICA components into “good” and “bad” by training data (HCP_hp2000.RData) (Griffanti et al., 2014; Salimi-Khorshidi et al., 2014). The bad components were regressed out from the 4D rs-fMRI data. The segmented terminal (start/end) regions of bundles were non-linearly registered to the MNI template. The gray matter (GM) regions containing these terminal regions of bundles were identified from the segmented GM volumes obtained from T_1_-weighted anatomical images using Freesurfer 6.0. The functional connectivity (FC) was generated based on Pearson correlation between the averaged rs-fMRI time series signals of the identified GM regions. The identified GM regions for terminal regions of each bundle are listed in Supplementary Table 2.

#### Statistical Analysis and Relationships With Cognitive Assessments

For all 65 cognitively normal and MCI subjects, the relationships of the amount of WMH volume, TC and FC with cognitive outcomes were analyzed by the multiple linear regression, while adjusting for age, sex, handedness, diagnosis status and years of education.

The tract-specific relationships of WMH, TC, and FC with multiple cognitive outcomes (MoCA and neuropsychological batter tests) were assessed by lasso linear regression. When performing linear regression with many variables that related to each other, it is difficult to interpret the model. The lasso regression provides a simpler model by automatically selecting important features (most relevant variables) in the model by the sparsity. In other words, this reduces a risk of detecting spurious associations. Thus, it is desirable for better interpretation of model with many variables. The sparsity is determined by the lasso (L_1_-norm) constraint. This type of regularization results in a sparse model by forcing the coefficients of some variables to be zero. i.e., irrelevant variables are not included in the final model. In this study, a set of tracts related to a cognitive outcome was determined.

In lasso regression analysis, independent variables were 50 tracts of WMH volume and covariables (age, sex, handedness, years of education and diagnosis) and dependent variable was each cognitive outcome. Thus, a total of 6 models were compared. The lasso regression analyses of TC and FC were tested with the same manner. In order to automatically determine the optimal regularization parameter (λ), i.e., *L*_1_-norm constraint, (1) 1,000 logarithmically spaced λ-values between 10^–5^ and 10^2^ were created, and the model was fit with these λ-values, (2) the maximal λ was determined when all coefficients (β) were zero, (3) the minimal λ was set to 0.001 * λ_max_, (4) a grid of 100 equally spaced points of λ on the logarithmic scale between the minimal and maximal λ-values was created, (5) the model was performed by fivefold cross-validation. This was repeated 1,000 times with randomly chosen training and test dataset. (6) The cross-validated mean squared error (MSE) was estimated and (7) MSEs from 1,000 repetitions were averaged. (8) The λ with the minimum MSE was chosen as the optimal λ. Supplementary Figure 2 demonstrates automated selection of the regularization parameter (λ) for the sparsity. Finally, the model was assessed using the optimal λ. Statistical significance of the identified set of tracts was determined by 20,000 permutations.

The mediation analysis was performed using Preacher and Hayes method (Preacher and Hayes, 2008) with the BRAVO toolbox^7^ to evaluate the causal relations between WMH and each cognitive measure through two-step pathway of TC—FC, through TC, and through FC. The direct pathway between WMH and each cognition was also assessed after controlling for the indirect effects. Nuisance variables of age, sex, handedness, years of education, and diagnosis were adjusted. The causal relation between WMH and TC through the pathway of AFD was also assessed. The statistical significance of the direct and indirect pathways was determined by 20,000 permutations.

## Results

Table 1 summarized the demographic and cognitive characteristics of the study participants. MOCA, attention, memory, language, and executive function were significantly different between cognitively normal and MCI subjects (*p* < 0.05), while visuospatial function showed no significant difference.

**TABLE 1:** Demography, cognitive measures, and neuroimaging measures by MRI.

	Cognitively normal (*n* = 49)	MCI (*n* = 16)

**Demography**		
Age (year)	67.1 ± 6.7	68.6 ± 12.2
Sex (fe/male)	35/14	9/7
Handedness (R/L)	48/1	16/0
Education (year)	16.9 ± 2.9	15.1 ± 3.2*
**Cognitive measures**		
MOCA^b^	27.1 ± 1.7	22.8 ± 2.4**
Attention^w^	26.9 ± 8.1	33.2 ± 10.8*
Executive^w^	63.4 ± 24.7	97.5 ± 42.3**
Memory^b^	8.0 ± 1.4	4.5 ± 2.7**
Language^b^	22.2 ± 5.7	18.2 ± 5.4*
Visuospatial^b^	21.1 ± 1.6	20.2 ± 1.7

For neuropsychological battery tests, higher score indicates worse performance for attention and executive functions^w^, while higher score indicates better performance for other neuropsychological tests^b^. Statistical difference between cognitively normal and MCI subjects, *p < 0.05; **p < 0.01.

**FIGURE 1:**
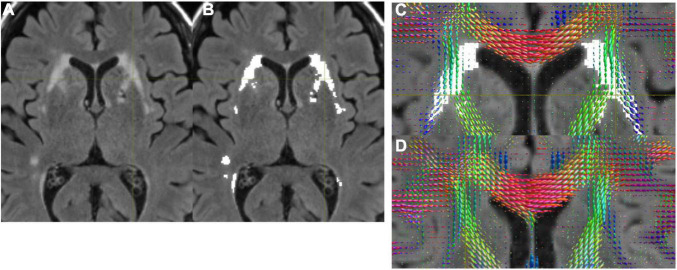
**(A)** A FLAIR image. **(B)** WMH was successfully segmented by the automated program. **(C)** FODs were overlaid on FLAIR with WMH. Altered FODs demonstrated in the WMH regions, compared to similar regions of a subject without WMH **(D)**.

Figure 1 shows structural MRI data from normal subjects of a 71-year-old female (Figures 1A–C), and a 68-year-old female (Figure 1D). Figures 1A–C show the presence of WMH, while Figure 1D does not. In Figure 1B, we show that the WMHs were successfully segmented from a FLAIR image (Figure 1A). The FODs in the WMH regions appeared altered (Figure 1C) compared to the similar regions in a subject without WMH (Figure 1D).

**FIGURE 2:**
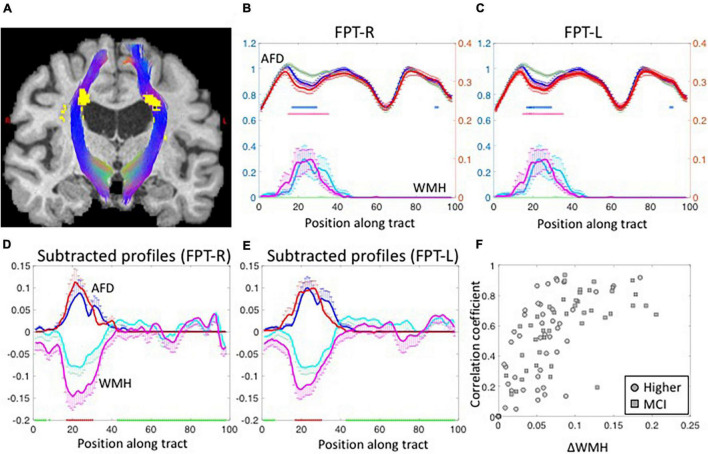
**(A)** The segmented fronto-pontine tracts (FPT) are overlaid on T1-weighted images. The tracts are passing through the segmented WMH. Red, Green and Blue represent the x, y, and z diffusion directions, respectively. **(B,C)** The group-averaged tract-profiles for right **(B)** and left FPT **(C)**. The AFD tract profiles (upper profiles) of higher-WMH (blue) and MCI-WMH (red) groups are statistically different with those of lower-WMH group (dark green) at the location of WMH in WMH tract profiles (lower profiles: light green color line for lower-WMH, light blue for higher-WMH, and magenta for MCI-WMH groups). Asterisk marks: *p* < 0.05 (blue for higher-WMH and pink for MCI-WMH groups, compared to lower-WMH group). Left axis (blue): AFD, right axis (red): WMH. Error bars: S.E.M. **(D,E)** The subtracted AFD profiles (blue lines) calculated by subtracting the lower-WMH group (green lines in **B,C**) from the higher-WMH group (blue lines in **B,C**). The subtracted AFD profiles between lower-WMH and MCI-WMH groups show as red lines. In the same way, the subtracted WMH profiles (cyan lines in **D,E**) were obtained from the lower- (green lines in **B,C**) and-higher WMH groups (cyan lines in **B,C**). The magenta lines are subtracted WMH profiles of lower-WMH from those of MCI-WMH groups. **(F)** The correlation coefficient values were calculated between subtracted AFD and subtracted WMH profiles, and plotted against the amount of WMH difference along the tract (ΔWMH was calculated by subtraction along the WMH profiles between less than 5% (green block on *x*-axis in **D,E**) and more than 50% (red block on *x*-axis in **D,E**) of the maximum in the profiles). The subtracted WMH profile were well correlated with subtracted AFD profiles at high ΔWMH. The correlation coefficient values were inverted for display purpose.

Figure 2A shows the WMH segmentations on left and right fronto-pontine tracts (FPT) overlaid on a T_1_-weighted image. The shapes of group-averaged tract-profiles for left and right AFD were similar (upper profiles in Figures 2B,C). The tract-profiles of AFD between lower-WMH and higher-WMH groups and between lower-WMH and MCI-WMH groups were statistically different mostly at the location of WMH (*p* < 0.05, blue asterisks for lower-WMH vs. higher-WMH groups, and red asterisks for lower-WMH vs. MCI-WMH groups in Figures 2B,C). In order to compare WMH and AFD changes, the tract-profiles of lower-WMH were subtracted from that of higher-WMH and MCI-WMH groups, respectively [blue line (= higher-WMH—lower-WMH) and red line (= MCI-WMH—lower-WMH) in Figures 2D,E]. WMH profiles were also subtracted with the same manner. The subtracted AFD profiles were highly correlated with the subtracted WMH profiles (Figures 2D,E). It indicates an increase in the local WMH on the tract is associated with a decrease in AFD. Similar results were observed in other tracts. Figure 2F shows the correlation coefficient values between subtracted WMH and AFD profiles against the amount of WMH on the tract. The correlation value between the amount of WMH and the AFD was high on tracts with larger WMHs, while this correlation was, of course, low on the tracts with smaller WMH because of little AFD changes due to small WMH.

**FIGURE 3:**
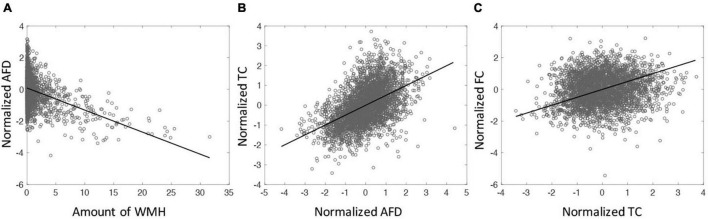
**(A)** The relationship between the amount of WMH on tractometry and normalized mean of AFD tract-profile (correlation coefficient (cc) = −0.41, *p* < 0.0001). **(B)** The relationship between normalized mean AFD and TC (cc = 0.50, *p* < 0.0001). **(C)** The relationship between TC and FC (cc = 0.12, *p* < 0.0001). All tracts across subjects are displayed. Each symbol indicates value for each tract of each subject. All 65 subjects are displayed.

The relationships among WMH, AFD, TC, and FC were assessed. Since the values of AFD, TC and FC are intrinsically different across tracts, these values were normalized (z-scored) across subjects for each tract. Figure 3A shows the amount of WMH is inversely related with the normalized AFD (*p* < 0.001). Figure 3B shows that AFD is linearly related with TC (*p* < 0.001). However, there is no significant relationship between TC and FC (*p* = 0.12, Figure 3C). With the mediation analysis, the indirect pathways of the AFD as a mediator between WMH and TC was statistically significant (*p* < 0.0001). Since the AFD and TC present similar quantities for WM microstructural changes and TC is more likely the result of AFD change on tract, we focus on TC for WM microstructure in the following analysis.

**FIGURE 4:**
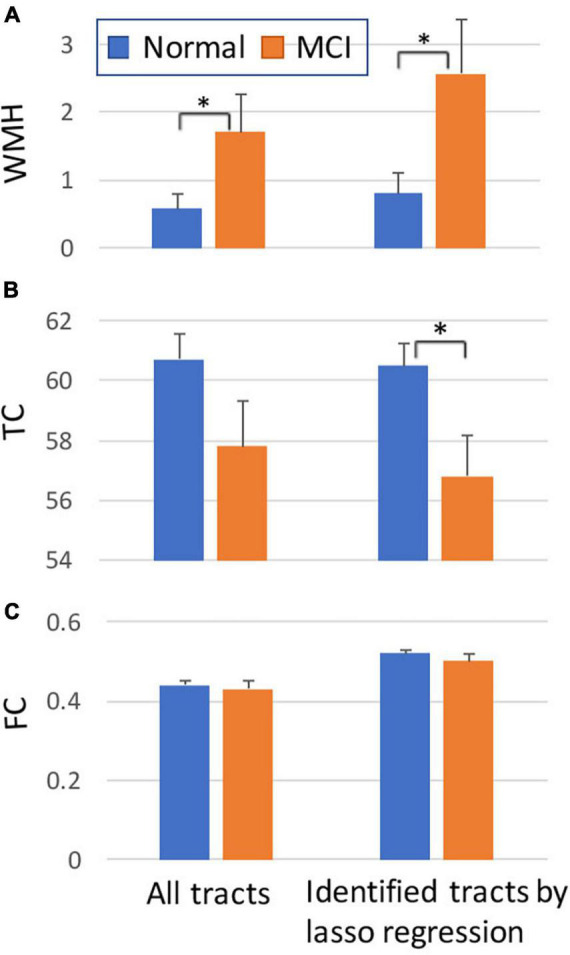
Comparisons between normal and MCI subjects for the amount of WMH volume **(A)**, TC **(B),** and FC **(C)** on tract. The graphs on the left were obtained from all tracts, while the graphs on the right were obtained from selected tracts by the lasso linear regression with cognitive measures (see Figure 5). *Y*-axis: arbitrary unit. Error bars: S.E.M. *p < 0.05.

For the average value of the 50 tracts, the amount of WMH volume was statistically different between normal and MCI subjects (*p* < 0.05), while the average values of TC and FC for all tracts were not statistically different (Figure 4, left). Note that the amount of WMH shown in Figure 4A is an arbitrary unit because it is a summation from tract profiles. The absolute WMH volumes were 2,266 ± 5,472 and 6,729 ± 8,645 mm^3^ for normal and MCI subjects, respectively (*p* < 0.05). The amount of WMH volume was significantly related to attention and executive functions [*p* < 0.0068 (= 0.05/6), with the Bonferroni correction], but not with memory, language, and visual functions (Table 2). The average values of TC and FC for all tracts were not statistically significant with all cognitive outcomes. These results may be caused by inclusion of tracts unrelated to cognitive change in the average value of all tracts.

**TABLE 2:** The relationships of the amount of WMH, TC, and FC with cognitive outcomes.

	WMH		TC		FC	
	All	Identified	All	Identified	All	Identified
MOCA	0.3787	0.3104	0.2090	0.0375	0.0295	0.0001*
Attention	0.0002*	0.0001*	0.1710	0.1070	0.6958	0.2110
Executive	0.0068*	0.0029*	0.0240	0.0049*	0.8696	0.4290
Memory	0.7256	0.7715	0.9493	0.7058	0.4951	0.5555
Language	0.2592		0.0159	3.9 × 10^–5^*	0.1367	0.0007*
Visuospatial	0.2543		0.3840		0.9890	

The p-values for the coefficient of each WMH, TC, and FC from the multiple linear regression are summarized. (all) The linear regression performed with values averaging from all tracts and (identified) values averaging from identified tracts. Blank in the identified column indicates that no tract is identified in the lasso regression. For identified tracts (see Figure 4). *p < 0.0068.

**FIGURE 5:**
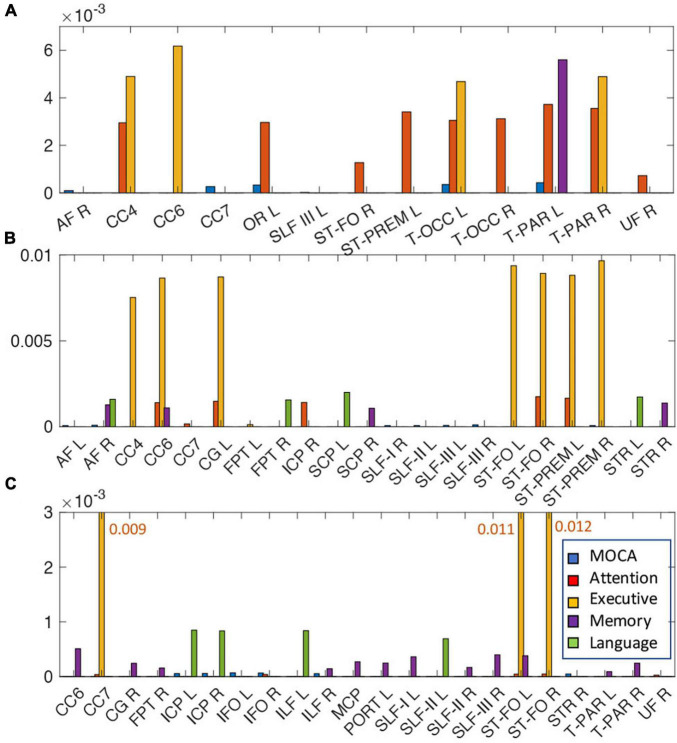
The relationships of the amount of WMH volume **(A)**, TC **(B)**, and FC **(C)** with various cognitive outcomes. The coefficient of WMH, TC, and FC for each cognitive outcome from the lasso linear regression with adjusting age, sex, handedness, diagnosis status and years of education as covariates. Statistically significant tracts are displayed (*p* < 0.0068). Since each cognitive outcome has different units of measurement, the coefficient of each cognitive outcome was divided by the average value of the cognitive outcome to normalize unit across multiple cognitive outcomes, and converted to absolute value for display purpose. For each cognitive outcome, the color of bar graph is displayed as blue for MOCA, red for attention, yellow for executive, purple for memory, and green for language. No tracts were related with visuospatial function. For FC **(C)**, since the bar graph of the execution function is much larger than that of other functions, it is displayed with a number next to the bar graph. The abbreviations of tract are listed in Supplementary Table 1.

Tract-specific relationships of WMH, TC, and FC with cognitive measures were analyzed using the lasso linear regression to find a set of tracts that best related to each cognitive measure. The regression coefficients of WMH, TC, and FC for each cognitive measure are shown in Figure 5. For WMH volume, tracts connected with thalamus [thalamo-parietal (T-PAR) and thalamo-occipital (T-OCC)], striatum [striato-fronto-orbital (ST-FO) and striato-premotor (ST-PREM)], and corpus callosum (CC4, CC6) tracts were mostly associated with attention and executive functions (Figure 5A). For TC, corpus callosum (CC4, CC6, CC7), cingulum (CG), and tracts connected to striatum (ST-FO and ST-PREM) were related to attention and executive function; arcuate fascicle (AF) and superior longitudinal fascicle (SLF) were related to MoCA; AF, CC6, superior cerebellar peduncle (SCP) and superior thalamic radiation (STR) were related to memory; AF, fronto-pontine tract (FPT), SCP, and STR were related to language (Figure 5B). For FC, many identified tracts were related to memory (CC6, CG, ILF, MCP, PORT, SLF, T-PAR, and ST-FO); ICP, IFO, ILF, and STR were related to MoCA; CC7 and ST-FO were related to attention and executive function; ICP, ILF and SLF were related to language (Figure 5C). No tract was associated with visuospatial function.

The WMH volume averaging from the identified tracts by lasso regression (shown in Figure 5A) showed greater differences between normal and MCI subjects with larger WMH volume (Figure 4A, right), compared to results from all tracts (Figure 4A, left). TC of identified tracts showed greater differences compared to that of all tracts and became statistically different between normal and MCI subjects (Figure 4B, right, *p* < 0.05). However, overall FC strength of identified tracts increased, compared to that of all tracts, but the differences between normal and MCI subjects were similar (Figure 4C, right). The overall relationships of WMH volume, TC, and FC with cognitive measures also became statistically more significant when the linear regression was performed by the identified tracts, compared to the results from all tracts (Table 2). With the identified tracts, TC was significantly related to executive function and language, and FC was significantly related to MoCA and language.

**FIGURE 6:**
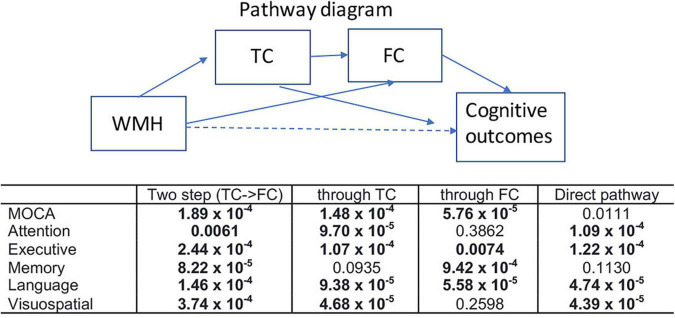
Results of mediation analysis are summarized for various cognitive outcomes (*p*-value for each pathway). Mediation analysis was tested for two step indirect path between WMH and each cognitive measure via TC—FC (two step), indirect pathway between WMH and each cognitive measure through TC (through TC), indirect pathway between WMH and each cognitive measure through FC (through FC), and the direct pathway between WMH and each cognition after controlling for the indirect effects. Nuisance variables of age, sex, handedness, years of education, and diagnosis were adjusted. Statistically significance results determined by 20,000 permutations are shown in bold (*p* < 0.0068).

Figure 6 shows the results of the mediation analysis, the statistical significance (*p*-values) of each pathway between WMH and multiple cognitive measures. The mediating pathway for each cognitive measure was different. The indirect pathways of FC between WMH and attention, and between WMH and visuospatial function were not significant, while those of TC between WMH and memory was not significant. Two step indirect pathways (TC—FC) between WMH and all cognitive measures were significant.

## Discussion

In the present study, higher WMH loads in WM tracts were locally matched with the reduced AFD, indicating WMH is associated with the microstructural alteration, which was related to decrease in TC. The amount of WMH volume in MCI subjects is larger than that in cognitively normal subjects and WMH is mostly associated with attention and executive function. TC of MCI subjects was statistically lower than that of normal subjects only in the identified tracts that was mostly associated with executive and language cognitive measures. Contrary to what we expected, there was no direct relationship between TC and FC and no difference between normal and MCI subjects in FC. It is noted that the FC in this study was analyzed for WM tract-based brain network, unlike cortical-oriented brain network (e.g., default mode network and dorsal attention network) in other studies. Tract-specific changes on WMH, TC and FC for each cognitive performance may explain that macro- and microstructural and functional changes are associated differently with each cognitive domain in a fiber specific manner. In addition, separated indirect pathways through TC and through FC were different depending on cognitive domain, indicating the relationship of WMH to each cognition may be associated by different paths of pathophysiology with a set of tracts.

### Advantage of Tract-Profile Approach

Fiber tracts in WM interconnect distant brain regions and play an essential role in overall brain connectivity. A voxel-level analysis may find structural changes, but they are not localized to abnormalities of a specific WM tract. Thus, segmentation of diffusion properties along a fiber tract provides localized information within individual’s WM bundles and analyzes differences in tracts of interest (Goodlett et al., 2009). Moreover, the tract-profile method suggested quantifying multiple bundle segments of tissue properties for the major WM connections in an individual’s brain as a function of arc length along the tract (Yeatman et al., 2012). The segmented tract-profile approach facilitates comparing the various shapes of the tracts between individuals as well as simplifying the data dimension. It helps to extract tissue properties of the major WM pathways from the areas containing morphological changes in an altered individual’s brain due to aging or disease progression. Thus, the tract-based approach has been shown prominent results when performing group comparison (Chamberland et al., 2019; Versace et al., 2019; Zhang et al., 2019). In addition, the different length of each fiber tract is difficult to compare across tracts. The equal segmentation of the tract is practically useful for comparisons between different fiber bundles with various lengths. The tract-profile approach is also not hampered by inconsistency in gyrification patterns across individuals and atrophy when comparing controls and disease groups because this method does not require anatomical information. Thus, it produces less errors in assessments of the WM properties of the tract.

In this study, we analyzed WM microstructural features by HARDI-derived measures with multi-shell multi-tissue CSD approach to overcome the non-specificity of FA in resolving complex fiber architecture. Despite the fact that diffusion-tensor metrics have well-characterized the voxel-wise microstructural features of WMH regions (Jones et al., 1999; Maniega et al., 2015), these are not well suited to properly characterize microstructural changes in AD- and aging-related studies (Jones et al., 2013; Fu et al., 2020). Misleading results from the diffusion-tensor model were observed in voxels containing more than one fiber population (e.g., at fiber bundle crossings) when comparing controls and AD patients (Mito et al., 2018). This misleading could affect most brain regions because the proportion of these multi-directional voxels is approximately 70–90% in the WM (Jones et al., 2013; Dell’Acqua and Tournier, 2019). In addition, the tractography obtained by FA could falsely start or stop in the WMH regions due to FA reduction in WMH (Ciccarelli et al., 2008), while the principal fODF direction is preserved in WMH regions, and therefore has no impact on the quality of tractography (Theaud et al., 2017). Thus, compared to the diffusion-tensor method (Fu et al., 2020), the fODF-derived approach is expected to better characterize the changes of specific fiber populations for WMH regions and detecting WMH-induced changes in WM microstructure.

### The Relationships Between Neuroimaging Measures and Multiple Cognitive Outcomes

Many studies have been shown that increased WMH volume is associated with poor cognitive performance. In the present study, WMH volume was related to attention and executive functions, but not with memory, language and visuospatial functions. This cognitive domain-specific variability agrees with previous results; larger WMH volume was related to executive function, but not memory (Dong et al., 2015); meta-analyses showed that among the cognitive domains, attention and executive function were specifically affected by WMH (Kloppenborg et al., 2014). In addition, WMH was found to affect both episode memory and execution, but its effect on episodic memory was mediated by executive function (Parks et al., 2011). These cognitive domain-specific variabilities by WMH may be caused by the spatial distribution of WMH or the order of different cognitive domain alteration progression. For example, the frontal WMH is mainly associated with attention and executive function, while parietotemporal WMH is related to memory (Burton et al., 2004; Lampe et al., 2019). Abnormalities in executive function typically precede deficits in language and spatiotemporal functions (Albert, 2011).

Both TC and FC averaging from the identified tracts become more significant with cognitive measures, compared to the results from all tracts (Table 2). The differences of WMH and TC between normal and MCI were also increased in the identified tracts (Figure 5). Thus, it can be explained that specific tracts are related with cognitive alterations. This agrees the previous study of rhesus monkeys that degeneration of myelin sheaths and loss of axons (nerve fibers) in several but not all fiber bundles were correlated with cognitive decline (Peters and Kemper, 2012). Subjects from mostly normal and early stage of cognitive decline conditions in this study may be another factor for tract-specific results. Since the progression of cognitive decline occurs slowly over a long period of time, the severe changes in TC and FC have not yet developed in many regions of the brain. Local microstructural change of AFD due to WMH may not be immediately affected to cause disruption of TC and cascading of further processing of cognitive deficits. A change above a certain threshold is required to have a causal effect (Boone et al., 1992), e.g., a certain amount of reduction in AFD on tract is required to cause a decrease in TC. In addition, cognitive reserve (e.g., education) may also play a significant role as a buffer in this process, causing high individual variability (Fernández-Cabello et al., 2016).

### Tract-Specific Relationship With Cognitive Measures

Identified tracts of each WMH, TC and FC were differently associated with performance in various cognitive domains. This agrees with the previous reports that tract-specific microstructures are associated with performance in certain cognitive domains (Ystad et al., 2011; Salami et al., 2012; Voineskos et al., 2012; Sasson et al., 2013; Cremers et al., 2016). The processing speed and executive function are commonly observed in relation with altered WM microstructural integrity. Except this, the results of reported tracts and related cognitive functions are heterogenous across the studies. This may be caused by discrepancies in the determination of tract extraction, neuropsychological battery tests, and focus of interest in the studies (Bolandzadeh et al., 2012). We analyzed different WM microstructural feature (i.e., TC) compared to the previous studies (mean or median of FA and MD in tract), thus, the direct comparisons of our findings with previous results are challenging. Our results showed that TC of posterior corpus callosum and tracts connecting striatum were most related to attention and executive function. Additionally, TC of thalamus-cerebellum, thalamus-superior frontal, and frontal-temporal connections were related to memory and language. Damages in a particular tract may be associated with a specific functional domain deterioration that the regions connecting the tract are responsible for. For example, the striatum interacts with the prefrontal cortex and contributes to executive function (Provost et al., 2015). The thalamus plays an important role in memory, executive functioning, attention and language (Van der Werf et al., 2003; Crosson, 2013), and exhibits a close partnership with other subcortical areas, especially the striatum (Wolff and Vann, 2019), while the frontal lobes support high-level cognition comprising executive skills and working memory.

On the other hand, the identified tracts in FC were mostly associated with memory function, but not overlapping with those identified in TC. This may be related to the fact that TC and FC in tract-based approach are not directly associated as shown in Figure 3. Deterioration in FC may occur in different cognitive domains through different path of mechanism, which may be independent with WMH-induced TC process. Overall, the identified tracts for WMH and TC are largely related with attention and executive function, while those for FC are mostly related to memory function. The mediation analysis showed the indirect path through TC was not significant between WMH and memory, while that through FC was not significant between WMH and attention and visuospatial function. It indicates that alterations in TC and FC may occur in different cognitive domains through different pathways, at least in aging or early stage of disease progression.

### Potential Limitations

We studied mostly normal subjects and relatively small number of MCI subjects. Thus, the pathology features of WMH-induced cognitive deficits may not be fully integrated in this study. However, abnormalities in WM are considered one of the earliest changes in the context of AD pathology in pre-dementia stages (Peters and Rosene, 2003; Gunning-Dixon et al., 2009; Nasrabady et al., 2018). Thus, our results may be helpful to understand early mechanism of cognitive decline. The connectivity results may vary depend according to different parcellation schemes for node, which obstruct the comparison of results across studies (Stanley et al., 2013). Regions (node) analyzed for FC and TC in this study need to be cautious when comparing them with other studies. In addition, larger increase in WMH burden may be associated with steeper cognitive decline over the same time period (Vannorsdall et al., 2009). However, the cross-sectional design of this study cannot assess these longitudinal changes. Thus, it implies that there is no answer about the temporality of alterations (progression or accumulation) of WMH and their direct relationships to alterations in brain connectivity and cognitive decline.

## Conclusion

The burden of WMH lesions contributing to local microstructural alterations is related to WM TC, but is not directly associated with FC. The specific tracts of WMH, TC, and FC were differently associated with changes in specific cognitive performances, and alterations in these identified tracts may involve in specific cognitive deficits. In addition, changes in each cognitive domain on WMH loads were mediated through different pathways of TC and FC. Thus, specific cognitive deterioration may be affected by alterations in a set of different tracts that are differently associated with macro- and microstructural, and function changes. Therefore, assessing the effects of WMH and its associated changes on tracts help for better understanding of the inter-relationships of the multiple pathological changes occurring in older adults that lead to cognitive impairment.

## Data Availability Statement

The datasets presented in this study can be found in online repositories. The names of the repository/repositories and accession number(s) can be found below: https://www.humanconnectome.org/study/connectomics-brain-aging-and-dementia.

## Ethics Statement

The studies involving human participants were reviewed and approved by the Human Research Protection Office, University of Pittsburgh. The patients/participants provided their written informed consent to participate in this study. Written informed consent was obtained from the individual(s) for the publication of any potentially identifiable images or data included in this article.

## Author Contributions

TK contributed to the research project conception, data analysis, and writing original draft. RR and BS contributed to the acquisition of the data. BS and JB contributed to cognitive classification from neuropsychological test. YC and Y-FC contributed statistical analysis. TK and JB contributed to the interpretation of the data. TK and JB revised. JB acquired funding. JD, TH, and AC contributed data curation. All authors revised the article critically for important intellectual content and approved the final version of the article.

## Conflict of Interest

The authors declare that the research was conducted in the absence of any commercial or financial relationships that could be construed as a potential conflict of interest.

## Publisher’s Note

All claims expressed in this article are solely those of the authors and do not necessarily represent those of their affiliated organizations, or those of the publisher, the editors and the reviewers. Any product that may be evaluated in this article, or claim that may be made by its manufacturer, is not guaranteed or endorsed by the publisher.

## Footnotes

1
https://www.humanconnectome.org/hcp-protocols-ccf-template

2
http://afni.nimh.nih.gov/afni/

3
http://fsl.fmrib.ox.ac.uk/fsl

4
https://www.mrtrix.org/

5
https://www.humanconnectome.org/software/hcp-mr-pipelines

6
https://fsl.fmrib.ox.ac.uk/fsl/fslwiki/topup

7
https://sites.google.com/site/bravotoolbox
