# Supplementary material for: Tract Specific White Matter Lesion Load Affects White Matter Microstructure and Their Relationships With Functional Connectivity and Cognitive Decline
Source: Front Aging Neurosci. 2022 Feb 2;13:760663. doi: 10.3389/fnagi.2021.760663 (PMC8848259; doi:10.3389/fnagi.2021.760663)
Supplement: Supplementary file 1 [file Data_Sheet_1.docx]

**Supplementary materials**

**Figure S1**. Classification of lower, higher WMH and MCI-WMH groups. The WMH volume of each subject is displayed. The lower WMH and higher WMH groups were selected from bottom 30% and top 30% WMH volume of control subjects (15/49 = 0.3), respectively. The MCI-WMH group was selected from MCI subjects whose WMH volume was larger than five standard deviations from the mean of the lower WMH group (green dash line). Green circles: lower WMH group, blue circles: higher WMH group, red circles: MCI group, gray circles: not used subjects for the group classification. The horizontal lines of corresponding color for each group indicate the mean of each group.


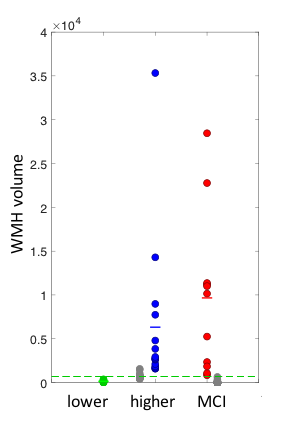


**Figure S2.** Automated selection of the regularization parameter (λ) for sparsity. The λ with minimum MSE was selected for the model (blue arrow). The number of coefficients (sparsity) was determined by λ (red circles). Lower λ value determines lower sparsity, while higher λ value results in higher sparsity (i.e. less number of coefficients remains in the model)


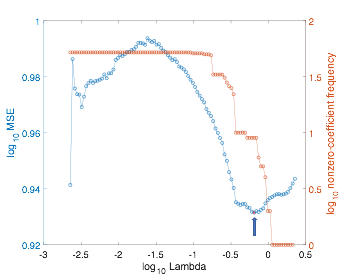


**Table S1**. The list of identified white matter tracts from the Tractseg program

|  | Tract name |
| --- | --- |
| 1 | AF (arcuate fascicle) left |
| 2 | AF right |
| 3 | ATR (anterior thalamic radiation) left |
| 4 | ATR right |
| 5 | CC (corpus callosum) 1 (rostrum) |
| 6 | CC2 (genu) |
| 7 | CC3 (rostral body (premotor)) |
| 8 | CC4 (anterior midbody (primary motor)) |
| 9 | CC5 (posterior midbody (primary somatosensory)) |
| 10 | CC6 (isthmus) |
| 11 | CC7 (splenium) |
| 12 | CG (cingulum) left |
| 13 | CG right |
| 14 | CST (corticospinal tract) left |
| 15 | CST right |
| 16 | FPT (fronto-pontine tract) left |
| 17 | FPT right |
| 18 | ICP (inferior cerebellar peduncle) left |
| 19 | ICP right |
| 20 | IFO (inferior occipito-frontal fascicle) |
| 21 | IFO right |
| 22 | ILF (inferior longitudinal fascicle) left |
| 23 | ILF right |
| 24 | MCP (middle cerebellar peduncle) |
| 25 | OR (optic radiation) left |
| 26 | OR right |
| 27 | POPT (parieto‐occipital pontine) left |
| 28 | POPT right |
| 29 | SCP (superior cerebellar peduncle) left |
| 30 | SCP right |
| 31 | SLF (superior longitudinal fascicle) I left |
| 32 | SLF I right |
| 33 | SLF II left |
| 34 | SLF II right |
| 35 | SLF III left |
| 36 | SLF III right |
| 37 | STR (superior thalamic radiation) left |
| 38 | STR right |
| 39 | UF (uncinate fascicle) left |
| 40 | UF right |
| 41 | T_PREM (thalamo-premotor) left |
| 42 | T_PREM right |
| 43 | T_PAR (thalamo-parietal) left |
| 44 | T_PAR right |
| 45 | T_OCC (thalamo-occipital) left |
| 46 | T_OCC right |
| 47 | ST_FO (striato-fronto-orbital) left |
| 48 | ST_FO right |
| 49 | ST_PREM (striato-premotor) left |
| 50 | ST_PREM right |

**Table S2.** Cortical (or subcortical) regions that contributed to the termination of tractography. The percentage shows how much of each area occupies in the termination area across subjects. Regions show up to 80% of occupancy. The regions with parentheses, which do not satisfy lateralization, were included for functional connectivity analysis.

|  | Tract name | GM begin % | GM end % |
| --- | --- | --- | --- |
| 1 | AF L | Inferior temporal L. 24.5 ± 2.4  Superior temporal L. 24.4 ± 2.1  Middle temporal L. 23.7 ± 2.0  (fusiform L.) 12.2 ± 1.5 | Rostral middle frontal L. 28.3 ± 3.3  Precentral L. 23.4 ± 2.6  Caudal middle frontal L. 14.5 ± 1.9  Pars opercularis L. 15.2 ± 1.5 |
| 2 | AF R | Inferior temporal R. 25.4 ± 2.6  Superior temporal R. 24.2 ± 2.8  Middle temporal R. 31.9 ± 2.2 | Rostral middle frontal R. 36.4 ± 3.1  Precentral R. 19.7 ± 2.6  Caudal middle frontal R. 12.5 ± 1.5  Pars triangularis R. 12.6 ± 1.6 |
| 3 | ATR L | Rostral middle frontal L. 57.1 ± 2.6  Pars triangularis L. 15.2 ± 2.0  Lateral orbitofrontal L. 11.3 ± 1.4 | Thalamus L. 90.3 ± 2.3 |
| 4 | ATR R | Rostral middle frontal R. 60.8 ± 3.6  Pars triangularis R. 16.7 ± 2.5  Pars orbitalis R. 12.1 ± 1.3 | Thalamus R. 90.7 ± 1.9 |
| 5 | CC1 | Medial orbitofrontal L. 51.9 ± 4.2  Lateral orbitofrontal L. 37.8 ± 3.9 | Medial orbitofrontal R. 52.1 ± 4.5  Lateral orbitofrontal R. 33.7 ± 4.8 |
| 6 | CC2 | Superior frontal L. 42.9 ± 2.0  Rostral middle frontal L. 20.0 ± 1.8  Pars triangularis L. 7.0 ± 0.9  (Lateral orbitofrontal L.) 7.8 ± 1.2 | Superior frontal R. 39.3 ± 2.3  Rostral middle frontal R. 35.2 ± 2.5  Pars triangularis R. 8.6 ± 1.2 |
| 7 | CC3 | Caudal middle frontal L. 56.3 ± 8.0  Pars opercularis L. 31.0 ± 5.4 | Caudal middle frontal R. 71.7 ± 9.9  Pars opercularis R. 18.1 ± 5.5 |
| 8 | CC4 | Precentral L. 91.4 ± 3.0 | Precentral R. 91.8 ± 7.6 |
| 9 | CC5 | Postcentral L. 54.8 ± 3.7  Paracentral L. 25.5 ± 3.4 | Postcentral R. 54.4 ± 3.5  Paracentral R. 26.7 ± 3.5 |
| 10 | CC6 | Superior parietal L. 20.4 ± 1.8  Precuneus L. 15.7 ± 1.3  Superior temporal L. 14.8 ± 1.3  Supramarginal L. 13.5 ± 1.4  Inferior parietal L. 12.5 ± 1.7  Middle temporal L. 10.0 ± 1.1 | Superior parietal R. 21.3 ± 2.1  Precuneus R. 15.0 ± 1.3  Superior temporal R. 13.1 ± 1.5  Supramarginal R. 12.9 ± 1.3  Inferior parietal R. 13.8 ± 2.1  Middle temporal R. 12.4 ± 1.1 |
| 11 | CC7 | Lateral occipital L. 33.9 ± 4.8  Lingual L. 19.3 ± 2.8  Cuneus L. 11.4 ± 1.6  Pericalcarine L. 10.7 ± 1.5  fusiform L. 13.0 ± 2.5 | Lateral occipital R. 40.3 ± 3.8  Lingual R. 16.4 ± 2.8  Cuneus R. 11.8 ± 2.1  Pericalcarine R. 12.1 ± 1.9  fusiform R. 10.1 ± 1.8 |
| 12 | CG L | Superior frontal L. 51.9 ± 2.9  Medial orbitofrontal L. 13.0 ± 1.8  Rostral middle frontal L. 11.0 ± 1.9  (Rostral anterior cingulate L.) 8.7±1.1 | Precuneus L. 49.7 ± 3.7  Posterior cingulate L. 16.0 ± 2.1  Isthmus cingulate L. 15.5 ± 2.4 |
| 13 | CG R | Superior frontal R. 59.1 ± 4.4  Medial orbitofrontal R. 9.1 ± 1.6  Rostral middle frontal R. 11.6 ± 2.1  (Caudal anterior cingulate R.) 8.8±1.3 | Precuneus R. 51.0 ± 3.2  Posterior cingulate R. 14.2 ± 2.6  Isthmus cingulate R. 14.2 ± 2.0  (Parahippocampal R.) 10.7 ± 1.2 |
| 14 | CST L | Precentral L. 92.7 ± 2.9 | Brain-Stem 97.0 ± 1.8 |
| 15 | CST R | Precentral R. 92.3 ± 7.5 | Brain-Stem 94.9 ± 2.6 |
| 16 | FPT L | Superior frontal L. 94.9 ± 2.1 | Brain-Stem 92.1 ± 1.9 |
| 17 | FPT R | Superior frontal R. 92.7 ± 2.7 | Brain-Stem 90.3 ± 2.2 |
| 18 | ICP L | Cerebellum L. 94.7 ± 1.6 | Brain-Stem 72.9 ± 14.4  Cerebellum L. 33.14 ± 10.4 |
| 19 | ICP R | Cerebellum R. 99.0 ± 0.7 | Brain-Stem 60.9 ± 15.4  Cerebellum R. 43.1 ± 12.5 |
| 20 | IFO L | Rostral middle frontal L. 35.0 ± 3.5  Lateral orbitofrontal L. 29.0 ± 2.7  Pars triangularis L. 14.5 ± 2.1  Pars orbitalis L. 10.0 ± 1.1 | Lateral occipital L. 43.9 ± 3.8  Lingual L. 23.6 ± 2.6  Cuneus L. 10.5 ± 1.2  Pericalcarine L. 10.3 ± 1.5 |
| 21 | IFO R | Rostral middle frontal R. 35.7 ± 4.0  Lateral orbitofrontal R. 29.9 ± 2.8  Pars triangularis R. 15.3 ± 2.3  Pars orbitalis R. 13.5 ± 1.2 | Lateral occipital R. 44.1 ± 3.0  Lingual R. 22.4 ± 2.1  Cuneus R. 11.5 ± 1.6  Pericalcarine R. 11.0 ± 1.5 |
| 22 | ILF L | Middle temporal L. 25.2 ± 5.7  Superior temporal L. 29.9 ± 5.7  Inferior temporal L. 18.8 ± 3.6  Fusiform L. 15.4 ± 3.2 | Lateral occipital L. 35.1 ± 5.3  Lingual L. 38.7 ± 4.5  Fusiform L. 15.1 ± 3.0 |
| 23 | ILF R | Middle temporal R. 32.1 ± 5.5  Superior temporal R. 25.8 ± 6.5  Inferior temporal R. 22.3 ± 4.4 | Lateral occipital R. 40.3 ± 5.6  Lingual R. 32.3 ± 4.5  Fusiform R. 15.2 ± 2.8 |
| 24 | MCP | Cerebellum L. 97.0 ± 1.0 | Cerebellum R. 99.3 ± 0.3 |
| 25 | OR L | Lateral occipital L. 61.9 ± 5.8  Lingual L. 15.0 ± 2.5  Pericalcarine L. 11.1 ± 2.4 | Thalamus L. 88.9 ± 2.4 |
| 26 | OR R | Lateral occipital R. 61.5 ± 6.3  Lingual R. 13.9 ± 2.5  Pericalcarine R. 11.8 ± 2.4 | Thalamus R. 84.9 ± 2.8 |
| 27 | POPT L | Superior parietal L. 51.3 ± 3.8  Postcentral L. 35.5 ± 3.3 | Brain-Stem 94.1 ± 1.7 |
| 28 | POPT R | Superior parietal R. 58.5 ± 4.9  Postcentral R. 31.1 ± 4.2 | Brain-Stem 91.3 ± 2.2 |
| 29 | SCP L | Cerebellum L. 93.2 ± 2.1 | Thalamus L. 88.4 ± 2.9 |
| 30 | SCP R | Cerebellum R. 96.4 ± 1.5 | Thalamus R. 85.5 ± 3.0 |
| 31 | SLF_I L | Superior frontal L. 63.1 ± 3.5  Caudal middle frontal L. 29.0 ± 3.8 | Superior parietal L. 84.8 ± 4.9 |
| 32 | SLF_I R | Superior frontal R. 40.5 ± 4.3  Caudal middle frontal R. 28.7 ± 3.6  Rostral middle frontal R. 27.0 ± 5.4 | Superior parietal R. 87.5 ± 6.5 |
| 33 | SLF_II L | Rostral middle frontal L. 35.4 ± 5.0  Superior frontal L. 31.6 ± 3.5  Caudal middle frontal L. 31.3 ± 4.4 | Inferior parietal L. 90.0 ± 4.9 |
| 34 | SLF_II R | Rostral middle frontal R. 48.4 ± 4.0  Superior frontal R. 24.5 ± 3.2  Caudal middle frontal R. 22.1 ± 2.7 | Inferior parietal R. 89.8 ± 4.3 |
| 35 | SLF_III L | Supramarginal L. 91.6 ± 4.4 | Pars opercularis L. 54.2 ± 4.9  Pars triangularis L. 33.4 ± 4.8 |
| 36 | SLF_III R | Supramarginal R. 91.4 ± 5.6 | Pars opercularis R. 32.9 ± 3.7  Pars triangularis R. 37.9 ± 4.4  (Pars orbitalis R.) 18.4 ± 3.1 |
| 37 | STR L | Paracentral L. 83.9 ± 7.6 | Thalamus L. 92.8 ± 2.1 |
| 38 | STR R | Paracentral R. 79.8 ± 8.0  (Precentral R.) 13.3 ± 2.7 | Thalamus R 88.7 ± 2.7 |
| 39 | UF L | Lateral orbitofrontal L. 63.8 ± 3.3  Pars orbitalis L. 17.5 ± 2.7 | Superior temporal L. 35.7 ± 7.2  Temporal pole L. 33.5 ± 5.8  Middle temporal L. 15.8 ± 4.3 |
| 40 | UF R | Lateral orbitofrontal R. 63.3 ± 3.3  Pars orbitalis R. 19.0 ± 2.5 | Superior temporal R. 37.2 ± 8.2  Temporal pole R. 26.7 ± 4.2  Middle temporal R. 22.2 ± 5.3 |
| 41 | T_PREM L | Caudal middle frontal L. 64.3 ± 10.4  Pars opercularis L. 26.1 ± 6.4 | Thalamus L. 88.4 ± 3.9 |
| 42 | T_PREM R | Caudal middle frontal R. 71.9 ± 10.3  Pars opercularis R. 16.6 ± 5.0 | Thalamus R. 88.7 ± 3.1 |
| 43 | T_PAR L | Superior parietal L. 31.9 ± 3.7  Inferior parietal L. 18.3 ± 2.7  Postcentral L. 19.5 ± 2.5  Precuneus L. 14.8 ± 2.1 | Thalamus L. 88.8 ± 2.4 |
| 44 | T_PAR R | Superior parietal R. 37.3 ± 4.9  Inferior parietal R. 24.1 ± 4.3  Postcentral R. 15.3 ± 1.9  Precuneus R. 14.6 ± 2.4 | Thalamus R. 87.21 ± 2.3 |
| 45 | T_OCC L | Lateral occipital L. 57.9 ± 5.4  Lingual L. 16.4 ± 2.5  Pericalcarine L. 11.5 ± 2.3 | Thalamus L. 88.6 ± 2.3 |
| 46 | T_OCC R | Lateral occipital R. 59.0 ± 5.6  Lingual R. 14.0 ± 2.6  Pericalcarine R. 12.3 ± 2.6 | Thalamus R. 85.2 ± 2.6 |
| 47 | ST_FO L | Lateral orbitofrontal L. 56.6 ± 3.8  Medial orbitofrontal L. 33.5 ± 3.6 | Putamen L. 38.1 ± 3.7  Caudate L. 38.9 ± 3.1  Lateral orbitofrontal L. 10.2 ± 3.2 |
| 48 | ST_FO R | Lateral orbitofrontal R. 52.7 ± 5.4  Medial orbitofrontal R. 32.0 ± 4.5 | Putamen R. 46.5 ± 5.4  Caudate R. 28.2 ± 3.2  Lateral orbitofrontal R. 11.4 ± 3.1 |
| 49 | ST_PREM L | Caudal middle frontal L. 74.1 ± 11.6  (Pars opercularis L.) 18.6 ± 6.9 | Putamen L. 43.8 ± 4.9  Caudate L. 27.2 ± 2.6  Pars opercularis L. 18.1 ± 4.1 |
| 50 | ST_PREM R | Caudal middle frontal R. 79.2 ± 12.3  (Putamen R.) 14.8 ± 4.4 | Putamen R. 40.2 ± 7.2  Pars opercularis R. 21.8 ± 5.0  Caudate R. 17.3 ± 3.2  (Pallidum R.) 12.1 ± 1.9 |
